# Effectiveness of Hypnosis for the Prevention of Anxiety During Coronary Angiography (HYPCOR study): a prospective randomized study

**DOI:** 10.1186/s12906-022-03792-x

**Published:** 2022-11-29

**Authors:** Laure Abensur Vuillaume, Charles Gentilhomme, Sandrine Weber, Nadia Ouamara, Julien Bayard, Mathieu Valla, Khalife Khalife, Christophe Goetz, Nazmine Guler

**Affiliations:** 1 Emergency Department, Mercy Hospital, Metz-Thionville Regional Hospital, Metz, France; 2 CUMP CHR Metz-Thionville, Metz, France; 3 Clinical Research Support Unit, Metz-Thionville Regional Hospital, Metz, France; 4 Department of Cardiology, Mercy Hospital, Metz-Thionville Regional Hospital, Metz, France

**Keywords:** Hypnosis, Coronarography, Emergency medicine

## Abstract

**Background:**

Coronary angiography is the gold standard for the diagnosis of coronary artery disease. This intervention is nevertheless a source of anxiety for the patient both by its discomfort and by the consequences linked to the discovery of potential diseases.

**Objectives:**

The aim of this study was to determine the effectiveness of hypnosis in reducing anxiety in patients undergoing coronary angiography.

**Methods:**

One hundred sixty-nine patients with planned coronary angiography and no history of coronary angiography were randomized to a hypnosis or control group. Patients in the hypnosis group underwent a hypnosis session with self-hypnosis posthypnotic suggestions, while those in the control group had a conversational interview with the hypnotherapist. The primary endpoint was pre-exam anxiety level assessed by the Spielberger State-Trait Anxiety Inventory (STAI-Y A).

**Results:**

Performing a hypnosis session did not result in a significant decrease in anxiety before the intervention. Age, high trait anxiety, high state anxiety the day before, and belief that hypnosis works in general were associated with increased anxiety before the procedure. No adverse events were reported after hypnosis. There was no statistically significant difference between the 2 groups for the occurrence of complications of the intervention.

**Conclusion:**

In this study, performing a hypnosis session before coronary angiography did not reduce the state of anxiety measured just before the intervention. In all cases, the hypnotic experience appears to be positive for the patient, encouraging further research efforts.

**Trial registration:**

The research protocol has been registered on the ClinicalTrials.gov registry (NCT02818101; 29/06/2016) and with the ANSM (IDRCB 2016-A00205-46; 02/02/2016).

## Background

Coronary angiography, a medical imaging technique used in cardiology to visualize the coronary arteries in the event of suspected coronary disease, has experienced spectacular growth over the past thirty years [1], in particular with the advent of radial puncture to replace femoral puncture. This interventional procedure is increasingly replacing gold standard surgical treatments. Percutaneous endoluminal revascularization techniques have also established themselves as a major treatment for acute and chronic coronary insufficiency [2, 3].

Coronary angiography is most often performed under local anesthesia [4]. Indeed, it is important to know during the intervention if the patient feels chest pain (which may indicate coronary injury). However, the absence of general anesthesia can be a source of anxiety for patients, which in itself can lead to adverse events [5, 6]. In addition, the procedure, which is usually painless, can become painful at different stages of the test: at the time of the puncture, during the progression of the probes along tortuous, spastic or highly calcified arteries, or during the placement of stent. In general, whether performed as an emergency or as a scheduled procedure, there is preoperative anxiety [7]. Anxiety can also affect the success of the procedure [8]. The diagnosis and its consequences can also be a source of stress for patients [9]. In the literature, preoperative anxiety is known as the most important and independent prognostic factor for mortality in patients over 70 years of age during cardiac surgery [10].

The State-Trait Anxiety Inventory (STAI) is a commonly used measure of trait and state anxiety (Spielberger, Gorsuch, Lushene, Vagg, & Jacobs, 1983). It can be used in clinical settings to diagnose anxiety and to distinguish it from depressive syndromes.

It has 20 items for assessing trait anxiety and 20 for state anxiety. State anxiety items include: “I am tense; I am worried” and “I feel calm; I feel secure.” Trait anxiety items include: “I worry too much over something that really doesn’t matter” and “I am content; I am a steady person.” All items are rated on a 4-point scale (e.g., from “Almost Never” to “Almost Always”). Higher scores indicate greater anxiety. The STAI is appropriate for those who have at least a sixth-grade reading level. STAI-Y A is commonly used to assess anxiety prior to cardiac catheterization combined with management techniques with the goal of reducing perioperative and pro-procedural anxiety. Music therapy is the most frequently evaluated method and it shows a significant decrease in STAI-Y A [11].

Several procedures have already been evaluated to reduce anxiety in patients before and during coronary angiography, including massage, music therapy, and aromatherapy [11–18]. Hypnosis is too one of the alternative medicines that has been evaluated. Hypnosis is a state of consciousness in which attention is focused and peripheral awareness reduced resulting in a high capacity to respond to suggestions [19]. This process can have a protective effect on pain, called hypnoanalgesia. It also reduces anxiety during therapeutic or diagnostic medical procedures [20–25]. A 2004 study of coronary angioplasty alone also compared hypnosis to drug sedation without significant results [26]. In addition, studies show that patients managed by hypnosis are more observant and the procedures are shorter by 45 min than for the control group; allowing greater operator comfort [27].

A recent review of the literature concluded that “the use of hypnosis during a surgical intervention or during a medical or interventional radiology act makes it possible to reduce the consumption of sedatives and/or analgesics during surgery [28, 29]. Thus, there is already some evidence for reducing the use of analgesics but not yet for reducing anxiety, especially before coronary angiography.

The objective of our study is to determine the effectiveness of hypnosis on anxiety before coronary angiography.

## Method

### Study population

The patients were recruited between March 20, 2016 and July 26, 2017 at the Metz Thionville Regional Hospital Center. Their hospitalization in cardiology was scheduled for the performance of a coronary angiography without emergency indication. The patients did not receive any systematic premedication for anxiolytic purposes. Patients who had undergone prior coronary angiography were not included, as past experiences may alter emotional state. Patients included were adults (≥ 18 years old) who underwent coronary angiography with or without stenting during the procedure. The exclusion criteria were: non-French speaking patients and/or cognitive disorders, and/or deaf or hard of hearing, and/or under legal protection, and/or psychiatric history of psychosis.

### Procedures

The day before the examination, a pre-inclusion visit was carried out by a cardiologist. An information document and a non-objection form were given to the patient. The patients included were randomly divided into two arms: the hypnosis group and the control group. Only the hypnotherapist was aware of the outcome of the draw. The cardiology department and the angiography technical platform teams were blind.

On the morning of the intervention, the patients in the hypnosis group underwent a hypnosis session with post-hypnotic suggestions in self-hypnosis to be carried out during the coronary angiography. The hypnosis session was the same for all patients with a script written for the study. The session lasted 15 min. It included visual induction and catalepsy techniques with the suggestion of a safe place where the patient treated the part of the body where he felt anxiety. A post-hypnotic suggestion was introduced at the end of the session, with a brief explanation of how to do it independently during coronary angiography with authorization to enter and exit hypnosis at their convenience to answer questions from caregivers. The purpose of the post-hypnotic suggestions was to facilitate self-hypnosis in the coronary angiography room so that the patient could manage their anxiety and pain. This session took place in the patient's room, before leaving for the coronary angiography department.

The hypnotherapists participating in the study were 4 emergency physicians, a pediatric nurse and an anesthetist nurse. All were trained in an institute recognized by the Francophone Confederation of Hypnosis and Brief Therapies and had at least 2 years of experience.

Patients in the control group had a conversational interview with one of the same hypnotherapists.

State anxiety and trait anxiety were measured in both groups the day before the procedure. State anxiety was measured again immediately before performing coronary angiography. Hemodynamic parameters (blood pressure and heart rate) were monitored (1) the day before surgery, (2) the morning of surgery in the patient's room, (3) immediately before coronary angiography, (4) immediately after coronary angiography and (5) the next day. Pain was assessed using a Visual Analogue Scale (VAS) the day before surgery, on arrival in the procedure room, on leaving the procedure room and the day after the intervention. Depending on the clinical situation, the operator could prescribe an anxiolytic (midazolam) and/or analgesic (paracetamol, morphine) treatment. The treatments and the occurrence of side effects were recorded in the file. At the end of the examination, the comfort of the operator was measured by a Likert scale of 1 to 5 points (1 meaning "very uncomfortable" and 5 the most comfortable). This score was collected by the coronary angiography nurse at the end of the procedure. The cardiologist was also asked to give his opinion on whether the patient belonged to the hypnosis or control group.

Patients were contacted at 1 month post-procedure to rate their overall satisfaction using a 1–5 point Likert scale (1 worst, 5 best). How satisfied are you with your management of coronary angiography?) Their opinion on the effectiveness of hypnosis on themselves as well as on the general population was collected.

### Endpoints

The primary endpoint was the patient's level of anxiety before coronary angiography using the SPIELBERGER’s inventory (STAI-Y A). The secondary endpoints were haemodynamic parameters, the use of anxiolytic or analgesic treatments and their respective dosages, pain assessment, operator comfort and patient satisfaction with their treatment.

### Supervision of the study

This randomized, single-blind, single-center study was designed by the principal investigator (N.G.) and methodologist (C.G.). The research protocol has been registered on the ClinicalTrials.gov registry (NCT02818101; 29/06/2016) and with the ANSM (IDRCB 2016-A00205-46; 02/02/2016). The Metz Thionville Regional Hospital Center sponsored and funded the trial. The research protocol was approved by the East-III Personal Protection Committee. The data was processed at the hospital's Clinical Research Support Platform. The methodologist (C.G.) checked the completeness and quality of the data, and performed statistical analyses.

### Statistical analysis

The sample size (85 patients per group) necessary to reach a power of 90% with an alpha risk of 5%, was calculated from the literature, which made it possible to estimate an expected score of 50 on STAI-Y A (standard deviation 10) for the control group and a clinically relevant difference of 5 points between the two groups 26,27.

The results are presented as median and ranges for qualitative data or as numbers and percentages for quantitative data. The hypnosis (experimental) and control groups were defined by intention to treat. The comparison between the two groups was carried out using Wilcoxon tests for the quantitative variables and Fisher's exact tests for the qualitative variables. A multivariate generalized linear model was used to identify factors that may influence the STAI Y A score before coronary angiography (age, sex, group, anxiety trait, anxiety state the day before, belief in the effectiveness of hypnosis in general and belief in the effectiveness of hypnosis for its own sake). The significance threshold was set at 5%. Bonferroni's corrections were applied for multiple tests where applicable. All analyzes were performed with SAS/STAT software version 9.4 (SAS Inst., Cary, NC).

## Results

**Fig. 1:**
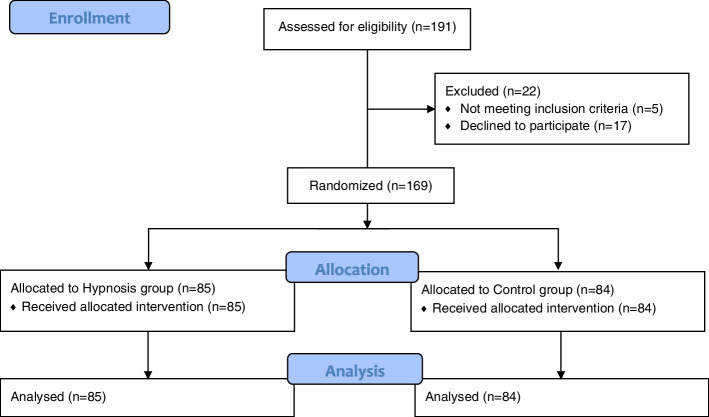
Patient Inclusion and Follow-up Diagram

A total of 169 patients were included in the analysis: 85 patients in the hypnosis group and 84 in the control group (Fig. 1). One patient initially randomized to the control group was excluded before the end of the study because he did not fulfill the inclusion criteria. Patient characteristics were similar between sthe two groups (Table 1), except for the response to the question "Do you think hypnosis works, in general?" to which more patients in the hypnosis group answered "no."

**Table 1:** Characteristics of the study patients at inclusion (day before coronary angiography)

Characteristic	Hypnosis group (*N* = 85)	Control group (*N* = 84)	P*
**Age (years)**	66 (44; 91)	68 (38; 88)	0.44
**Gender (F)**	27 (32)	30 (36)	0.63
**Believes that hypnosis works in general**			
** Yes**	59 (69)	62 (74)	0.03**
** No**	19 (22)	8 (10)	
**No opinion**	7 (8)	14 (17)	
**Believes that hypnosis works on himself**			
** Yes**	34 (40)	32 (38)	0.74
** No**	16 (19)	20 (24)	
** No opinion**	35 (41)	32 (38)	
**Heart rate (bpm)**	76 (49; 148)	72 (50; 177)	0.15
**Systolic blood pressure (mmHg)**	142 (93; 182)	148 (101; 192)	0.08
**Diastolic blood pressure (mmHg)**	77 (39; 109)	78 (40; 111)	0.79
**Pain (visual analog scale)**	0 (0; 5)	0 (0; 2)	1
**State anxiety (STAI-Y A)**	49 (37; 70)	49 (35; 73)	0.84
** In women**	50 (37; 65)	49 (35; 73)	0.74
** In men**	49 (39; 70)	49 (39; 73)	0.61
**Trait anxiety (STAI-Y B)**	47 (35; 64)	48 (27; 80)	0.96
** In women**	43 (36; 64)	46 (27; 80)	0.93
** In men**	48 (35; 64)	48 (31; 68)	0.86

Data are presented as median (min; max) or number (percentage)

^*^Wilcoxon or Fischer’s exact tests

^**^p < 0.05

**Fig. 2:**
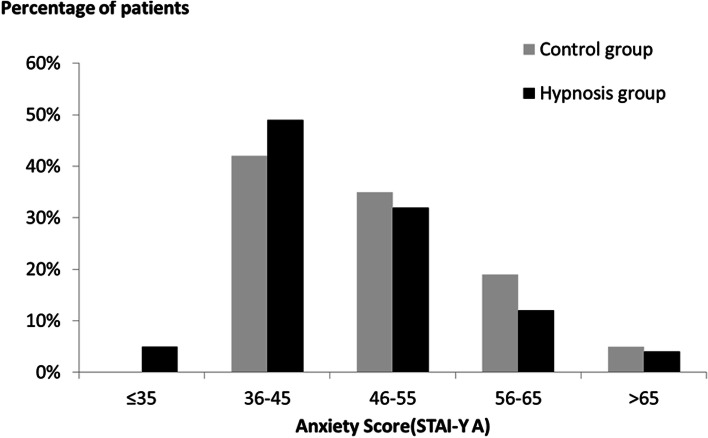
Comparison of anxiety score distributions (STAI-Y A) immediately before coronary angiography between the Hypnosis group (*N* = 85) and the Control group (*N* = 84)

The STAI-Y A immediately before coronary angiography was 45 (range: 35–73) in the hypnosis group and 46 (36–74) in the control group (Table 2). This difference was not statistically significant. The distribution of scores in the two groups is shown in Fig. 2. Midazolam was used in 9 patients (11%) in the hypnosis group versus 14 patients (17%) in the control group, this difference was not significant (Table 2). There was no significant difference between the 2 groups regarding the occurrence of adverse events, pain, operator satisfaction, and the operator's opinion of whether the patient belonged to the hypnosis group or the control group (Table 2).

**Table 2:** Comparison of the process and consequences of coronary angiography between the Hypnosis and Control groups

Characteristic	Hypnosis group (*N* = 85)	Control group (*N* = 84)	P*
**Use of Midazolam**	9 (11)	14 (17)	0.27
**Use of I-V paracetamol**	1 (1)	2 (2)	0.62
**Use of morphine**	1 (1)	2 (2)	0.62
**Operator believes that the patient is under hypnosis**			
** Yes**	54 (64)	50 (60)	0.85
** No**	22 (26)	25 (30)	
** No opinion**	9 (11)	9 (11)	
**Pain before the procedure (visual analog scale)**	0 (0; 8)*	0 (0; 7)*	0.95
** VAS ≥ 4**	5 (6)	7 (8)	0.57
**Pain after the procedure**	0 (0; 6)*	0 (0; 7)*	0.50
** VAS ≥ 4**	1 (1)	3 (4)	0.37
**Pain the day after the procedure**	0 (0; 6)*	0 (0; 3)*	0.42
** VAS ≥ 4**	3 (4)	1 (1)	0.62
**Complications**	8 (9)	11 (13)	0.48
** Vasovagal episode**	4 (5)	4 (5)	0.99
** Hematoma**	4 (5)	5 (6)	0.75
** Cutaneous allergy**	1 (1)	1 (1)	0.99
** Anaphylactic shock**	1 (1)	0 (0)	0.99
** Ventricular fibrillation**	0 (0)	1 (1)	0.50
**State anxiety (STAI-Y A) immediately before the procedure**	45 (35; 73)	46 (36; 74)	0.23
** In women**	44 (35; 65)	44 (36; 69)	0.53
** In men**	46 (39; 73)	48 (39; 74)	0.21
**Operator satisfaction**			
** Yes (greater than 4/5)**	58 (68)	59 (70)	0.99
** No (less than or equal to 4/5)**	18 (21)	17 (20)	
** No opinion**	9 (11)	8 (10)	

Data are presented as median (min; max) or number (percentage) *Wilcoxon or Fischer’s exact tests

In multivariate analysis (Table 3), the state of anxiety before coronary angiography was shown to decrease with age (*p *= 0.001) and with the belief that hypnosis works in general (*p* = 0.048). It has been shown to increase with trait anxiety (*p* = 0.03) and with state anxiety the day before (*p* < 0.0001). Gender and the belief that hypnosis works on himself were not significantly associated. An interaction between hypnosis group and beliefs about hypnosis was tested and was not significant (*p* = 0.10).

**Table 3:** Multivariate analysis (generalized linear model) of factors suspected of influencing state anxiety (STAI-Y A) prior to coronary angiography (*N* = 169, R^2^ = 0.47, *p* < 0.0001)

Characteristic	Parameter	Standard Deviation	*P*
**Hypnosis**	1.48	0.95	0.12
**Age (years)**	-0.16	0.05	0.001*
**Gender (F)**	-1.40	1.02	0.17
**Trait anxiety (STAI-Y B)**	0.13	0.06	0.03*
**State anxiety the day before (STAI-Y B)**	0.48	0.07	< 0.0001*
**Believes that hypnosis words on self**	-1.75	1.07	0.10
**Believes that hypnosis works in general**	-2.36	1.19	0.048*

^*^p < 0.005

Telephone follow-up at 1 month showed no difference in patient satisfaction with their care (Table 4). In contrast, more patients in the hypnosis group responded that they thought hypnosis was effective on them compared to the control group (*p* = 0.003).

**Table 4:** Follow-up on D30 (by telephone)

Characteristic	Hypnosis group (*N* = 35)	Control group (*N* = 35)	P*
**Overall satisfaction of care management**			
** Yes (greater than 4/5)**	24 (69)	28 (80)	0.99
** No (less than or equal to 4/5)**	11 (31)	7 (20)	
**Believes hypnosis works in general**			
** Yes**	28 (80)	27 (77)	0.99
** No**	4 (11)	4 (11)	
** No opinion**	3 (9)	4 (11)	
**Believes that hypnosis works on themselves**			
** Yes**	28 (80)	19 (54)	0.03**
** No**	7 (20)	9 (26)	
** No opinion**	0 (0)	7 (20)	

Data are presented as numbers (percentage)

^*^Fischer’s exact tests with Bonferroni correction for multiple testing

^**^p < 0.005

**Fig. 3:**
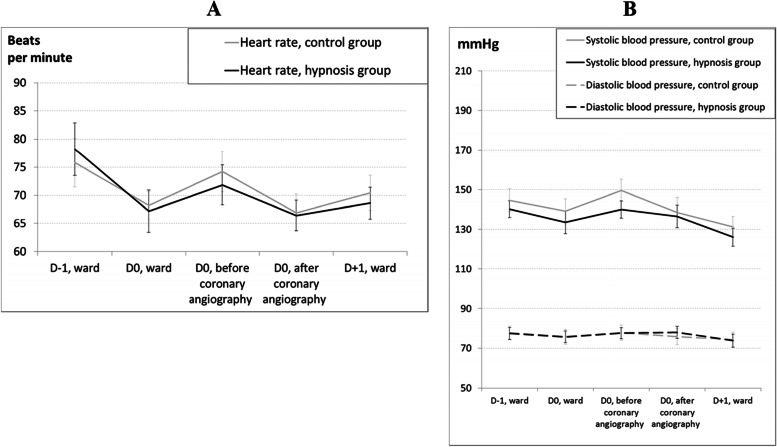
**A** and **B** Heart rate and blood pressure evolution in the hypnosis (*N* = 85) and control (*N* = 84) groups. Error bars represent 95% confidence intervals. No significant differences exist between groups at the different time points

For hemodynamic parameters, heart rate was identical in both groups (Fig. 3A). Systolic blood pressure appeared slightly lower in the hypnosis group, but the differences were not statistically significant (Fig. 3B). Diastolic blood pressure showed no difference between the groups.

## Discussion

To our knowledge, our study is the first to date to evaluate hypnosis by STAI before coronary angiography. It shows that carrying out a hypnosis session with self-hypnosis suggestions before coronary angiography did not result in a significant drop in the state anxiety score. With a median score of 45 in the hypnosis group and 46 in the control group, the anxiety levels of the patients included could be considered “average” [30]. These values ​​are higher than the norms reported for patients in medical stress situations such as patients hospitalized for various organic diseases, or undergoing medical examinations or surgery [30]. However, our results are in agreement with those of Lang et al., for patients hospitalized for a biopsy under hypnosis with suspicion of breast cancer or to perform a transesophageal ultrasound examination [21, 31]. The anxiety state score before the intervention, with alternative medicine, is on average comparable to that observed in our study [11, 14–17].

The lack of effectiveness of hypnosis in our study may be due to the timing of the assessment. Indeed, due to the constraints of the coronary angiography procedure, anxiety could not be measured during the procedure. It may also be due to the choice of the evaluation method. Indeed, if hypnosis is effective against anxiety, its statistical proof seems to be provided by an evaluation throughout the intervention and not in the moments preceding it [21, 23, 32, 33]. The patients were subjected to the Spielberger’s inventory on their arrival in the coronary angiography room, therefore before initiating a process of self-hypnosis. The evaluation carried out here therefore probably evaluates the effects of morning management and not the effectiveness of hypnosis during coronary angiography. This is in agreement with the results reported in several randomized clinical trials using STAI and involving interventional medicine showing a decrease in anxiety during an invasive examination but without significant difference in pre-interventional or during the first stages of the procedure. Lang et al., who also used the Spielberger inventory, found no significant difference in state anxiety scores before the procedure as well as during the first 0–15 min time interval [33]. A simple numerical rating scale is more sensitive to hypnosis-induced changes in anxiety [21, 32, 33]. Other scales, such as the Beck Anxiety Inventory or the Hamilton Anxiety Scale, also lead to the same conclusion [23]. These results therefore raise the possible interest of hypnosis during pre-coronary angiography since hypnosis showed a clear superiority compared to the groups receiving other additional measures such as "structured attention" or empathy [21, 32].

However, the main obstacle to the use of these techniques remains skepticism, maintained by the lack of scientific evidence, as it is difficult to measure the effectiveness of hypnosis or self-hypnosis, but also by the pejorative connotation disseminated, among others, by the entertainment world [34]. If self-hypnosis seems to be a promising tool for adapting to situations felt difficult (as demonstrated by Peter C. Keil in 2018 on chronic pain in hospitalized patients [35], its effectiveness must be objectively measured by standardized measures to expand its use and teaching. Our study found that patients' belief that "hypnosis works in general" prior to coronary angiography significantly influenced pre-intervention anxiety, regardless of the group to which they were assigned. This raises two important points: on the one hand, the beliefs and opinions of the patient vis-à-vis hypnosis and, on the other hand, the “hypnosis” label of the study. The general public develops ideas and expectations from different sources, including about medical care [36]. The cognitive model of hypnosis explains that variables such as participant motivation, beliefs or expectations are keys for patients' hypnotic receptivity [37, 38]. According to some authors, the reinforcement of these variables makes it possible to facilitate hypnotic reactivity [39]. On the other hand, Gandhi and Oakley highlighted the impact of the “hypnosis” label, whose title alone plays an anxiolytic role [40].

As observed by Dufresne et al., our study shows that one month after treatment, patients' opinions were influenced by the hypnotic experience [41]. This difference between the two arms of the study may suggest a use of self-hypnosis in the experimental group during the procedure. In any case, the improvement of the opinion regarding the hypnosis represents a positive reinforcement for the subsequent management. This hypothesis will require an evaluation of the effectiveness of hypnosis in patients who have previously benefited from this approach in the same procedure to confirm this hypothesis.

### Study limitations

Our study has several limitations. First, it is monocentric and therefore there is a risk of bias of non-representativeness of practices and population. However, clinical activity within Mercy Hospital's cardiology department is significant, with 4,500 coronary angiograms and 2,314 percutaneous coronary angioplasties performed in 2016. In addition, the study included the intervention of 6 different cardiologists and was also carried out with the intervention of 6 health personnel qualified in hypnosis. Therefore, given the number of patients and operators, we believe that our results can be extrapolated to other centers.

Second, our study could not be double-blind because the hypnotherapist had to know whether to perform a hypnosis session or an interview with the patient. In order to avoid any follow-up bias, it would seem appropriate that the intervention in the control group not be limited to a simple interview with the hypnotherapist but also by another unconventional technique.

Third, pain assessment was only performed at peri-procedural times. It is therefore possible that such an assessment was not suitable since literature reviews highlight the positive effects of hypnosis on pain during medical or surgical procedures and often find no difference in anxiety before the procedure as in our study [21, 22, 32, 33, 42–44]. Therefore, it would have been more appropriate to assess pain by VAS during the procedure. It would also have been interesting to assess whether the patient had used self-hypnosis during the intervention by questioning him afterwards. These elements will have to be taken into account in another study. Finally, if none of our patients received specific premedication, the patients' usual treatment was not defined as an exclusion criterion. Under these conditions, patients on anxiolytics (benzodiazepines, antidepressants, etc.) could be included, which may induce a bias. However, these practices are usual in the coronary angiography center in which the study was conducted and we did not wish to modify them.

## Conclusion

In the present study, performing a hypnosis session upstream of a coronary angiography, with posthypnotic suggestions in self-hypnosis to be performed during the procedure, failed to reduce the anxiety state measured immediately before the procedure, compared to a simple conversational interview with the hypnotherapist. Factors associated with lower state anxiety before the procedure were age, trait anxiety, and believing that hypnosis is an effective technique in general.

## Data Availability

The datasets used and/or analyzed during the current study available from the corresponding author on reasonable request.
